# The Role of Lactic Acid Bacteria in Meat Products, Not Just as Starter Cultures

**DOI:** 10.3390/foods13193170

**Published:** 2024-10-06

**Authors:** Kayque Ordonho Carneiro, Gabriela Zampieri Campos, João Marcos Scafuro Lima, Ramon da Silva Rocha, Manuela Vaz-Velho, Svetoslav Dimitrov Todorov

**Affiliations:** 1 ProBacLab, Laboratório de Microbiologia de Alimentos, Departamento de Alimentos e Nutrição Experimental, Food Research Center (FoRC), Faculdade de Ciências Farmacêuticas, Universidade de São Paulo, São Paulo 05508-000, SP, Brazil; kayqueordonho@usp.br (K.O.C.); gabriela.zampieri.campos@usp.br (G.Z.C.); joao.marcos.lima@usp.br (J.M.S.L.); 2 Laboratório de Microbiologia de Alimentos, Departamento de Alimentos e Nutrição Experimental, Food Research Center (FoRC), Faculdade de Ciências Farmacêuticas, Universidade de São Paulo, São Paulo 05508-000, SP, Brazil; ramonrocha@usp.br; 3 CISAS—Center for Research and Development in Agrifood Systems and Sustainability, Escola Superior de Tecnologia e Gestão, Instituto Politécnico de Viana do Castelo, 4960-320 Viana do Castelo, Portugal; mvazvelho@estg.ipvc.pt

**Keywords:** fermented foods, meat products, bioactive compounds, antimicrobial, bacteriocins, health benefits

## Abstract

Lactic acid bacteria (LABs) are microorganisms of significant scientific and industrial importance and have great potential for application in meat and meat products. This comprehensive review addresses the main characteristics of LABs, their nutritional, functional, and technological benefits, and especially their importance not only as starter cultures. LABs produce several metabolites during their fermentation process, which include bioactive compounds, such as peptides with antimicrobial, antidiabetic, antihypertensive, and immunomodulatory properties. These metabolites present several benefits as health promoters but are also important from a technological point of view. For example, bacteriocins, organic acids, and other compounds are of great importance, whether from a sensory or product quality or a safety point of view. With the production of GABA, exopolysaccharides, antioxidants, and vitamins are beneficial metabolites that influence safety, technological processes, and even health-promoting consumer benefits. Despite the benefits, this review also highlights that some LABs may present virulence properties, requiring critical evaluation for using specific strains in food formulations. Overall, this review hopes to contribute to the scientific literature by increasing knowledge of the various benefits of LABs in meat and meat products.

## 1. Introduction

**Figure 1:**
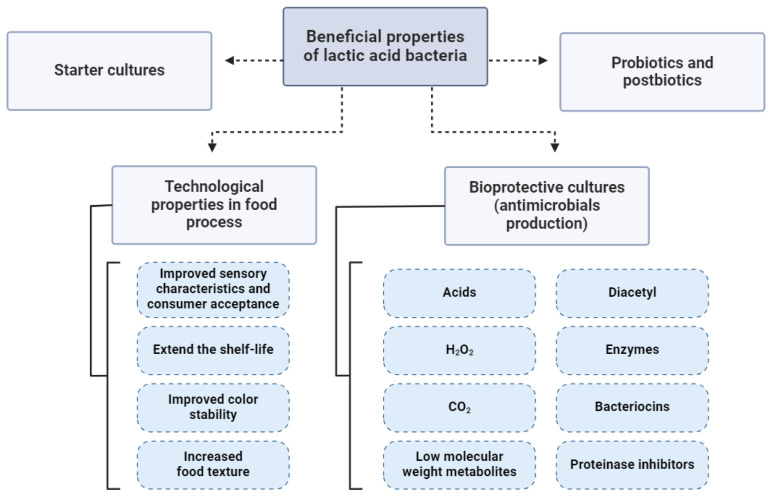
Beneficial properties of lactic acid bacteria, from the essential driver of the fermentation processes and contributors to the technological properties of the final products to important players in the control of spoilage and pathogens and providing probiotic (and postbiotics) benefits for the consumers.

Lactic acid bacteria (LABs) are one of the most studied (non-taxonomical) groups of microorganisms concerning their beneficial properties and include representatives from different genera, such as *Lactobacillus*, Lactococcus, Pediococcus, Streptococcus, Enterococcus, Carnobacterium, Tetrogenococcus, Leuconostoc, and *Oenococcus*. The main characteristics of these groups are the production of lactic acid as the final metabolite of carbohydrate metabolisms, Gram-positive and catalase-negative rods or cocci as morphology and not spore forming. Moreover, in 2020 Zheng et al. [1] suggested an enormous reclassification for former genera *Lactobacillus and Leuconostoc* and suggested 25 new genera. Furthermore, Todorov et al. [2] suggested standardized abbreviations for all the new suggested genera. The LAB groups have, for centuries, scientifically proven the beneficial role in the preparation of fermented food products, playing an essential role in technological and biopreservative processes, improving human health when applied as probiotics [3], being considered as GRAS (generally recognized as safe), and being applied in several biotechnological processes [4] (Figure 1). On the other hand, several LABs have been well evaluated as human and other animal pathogens (opportunistic and effective), such as several representatives from the genera *Streptococcus* and *Enterococcus*, which are notable examples of pathogenic LABs [5,6]. Moreover, with the development of research tools and biomolecular approaches, even more reports have provided evidence that some species/strains that have been considered safe need to be applied with high preclusions since some virulence properties can be associated with them.

Acidification is one of the most important factors to be considered for the selection and isolation of LABs to be used as starter cultures in food products [7]. In this context, the main application of LABs in meat products is as a starter culture, given the technological and functional benefits that they generate in the product [8]. These microorganisms are added to meat product formulations and, soon after the start of their metabolism, significantly modify the sensory characteristics of the products, mainly flavor, aroma, and texture, and contribute to safety via the production of antimicrobial peptides and other antagonistic metabolites. Moreover, the production of acid from the metabolism of starter cultures also reduces the pH, which generates important effects on product quality, favoring the coagulation process and the stability and development of the intended color [9]. In addition, they ensure greater safety and stability of some compounds during shelf life [10].

This review aims to provide a comprehensive review of the characteristics, beneficial properties, and potential applications of LABs in meat and meat products and explore the production of bioactive compounds and other important metabolites and their potential application to improve food quality and health-promoting properties.

## 2. Bioactive Peptides

**Figure 2:**
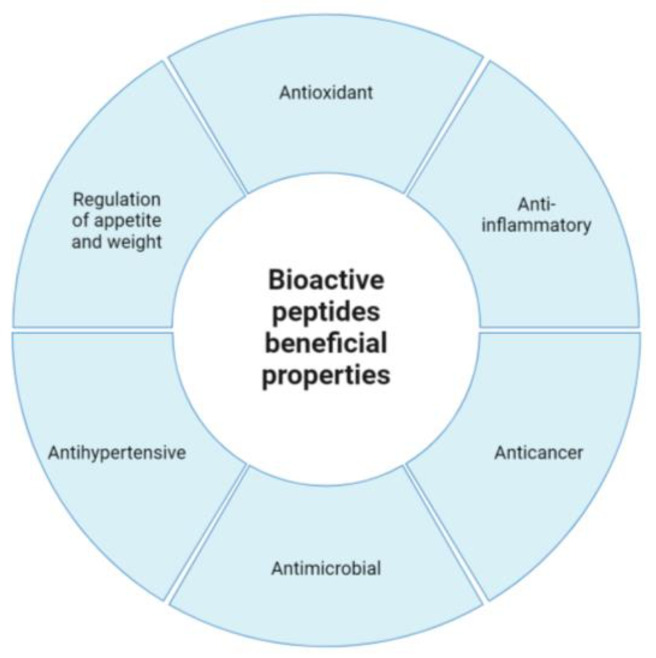
General areas for effects of the bioactive peptides produced by lactic acid bacteria.

LABs are scientifically and industrially recognized for their biokinetic ability and physiological properties to produce an enormous variety of bioactive compounds during fermentation. Some of the main bioactive compounds produced by LABs are a sizable variety of functional peptides originated by the biotransformation of the naturally presented proteins in the food matrixes that can play different beneficial properties, including antimicrobial, antihypertensive, and immunomodulatory [11], and contribute to the quality, safety, and health-promoting properties of fermented foods. Bioactive peptides are typically composed of 2 to 20 amino acid residues and have a range of health benefits such as: (A) antioxidative roles associated with the fact that they can scavenge present free radicals and play a role in the reduction of the oxidative stress, which are conditions well associated with some chronic diseases such as cancer and cardiovascular diseases; (B) anti-inflammatory properties are characteristic of bioactive peptides, related to their ability to participate in the modulation of the body’s inflammatory response, a role beneficial in clinical conditions such as arthritis and other inflammatory diseases; (C) anticancer properties for some bioactive peptides have been suggested in relation to their properties for the inhibition of the proliferation of cancer cells and related to thus, such as metabolites may contribute to cancer prevention and treatment [12]; (D) antimicrobial properties, possibly one of the most studied properties for LABs, where bioactive peptides can be formed by the biotransformation of the substrate proteins or produced by the ribosomal machinery of the LABs (a group of polypeptides that will be discussed separately). Antimicrobial bioactive peptides can actively inhibit the growth of harmful microorganisms (and even kill them), which suggests they could be a potential alternative to traditional antibiotics. Immunomodulation properties of bioactive peptides can contribute to enhancing or suppressing the immune response, which can play a role in managing autoimmune diseases and allergies [12]; (E) antihypertensive beneficial properties are well associated with fermented food products and are mapped as effective as some bioactive peptides. These peptides can improve processes in lowering high blood pressure, which is a major risk factor for heart disease [13]; (F) involvement in the regulation of appetite and weight, where specific bioactive peptides can be involved in influencing the production of some hormones and, as a consequence, participate in the control of appetite and satiety, potentially aiding in weight management; the improvement in diabetes and blood glucose regulation can be a link to the beneficial role of some bioactive peptides [14]. Bioactive peptides need to be clearly differentiated from beneficial peptides produced by the ribosomal machinery of LABs, since they (bioactive peptides) are derived from proteins, part of the substrate used for the biological function of LABs, and part of the various food sources, including meat, dairy, plants, and marine organisms. However, as part of the fermented food product, bioactive peptides play an essential role in beneficial properties and have potential application in the development of functional foods, nutraceuticals, and pharmaceuticals [12,15] (Figure 2).

In principle, every food containing protein in its composition and exposed to some fermentation process can be considered a potential bioactive peptide source. Between them, fermented meat products have been shown to release peptides with antioxidative and antimicrobial activities. Moreover, dairy products (fermented milk and cheeses) are excellent sources of bioactive peptides, particularly those with antihypertensive properties, well suggested for centuries by traditional medicine [16]. Egg proteins can be hydrolyzed to produce peptides that have various health benefits. Aquatic creatures, including various fishes, contain peptides that may have antihypertensive and antioxidative effects [17]. Grains such as oats, rice, sorghum, barley, and wheat; legumes, including beans, peas, and lentils; and certain vegetables and fruits or nuts are plant sources that contain peptides that can contribute to health [18]. All these food sources, especially when fermented, can actively contribute to harnessing the health benefits of bioactive peptides through diet.

## 3. Exopolysaccharides

Exopolysaccharides (EPSs) are extensively studied groups of microbial metabolites with numerous beneficial properties and are subject to particular interest. EPSs are long-chain sugar molecules produced by LABs (and other microbial groups) that can play a role in technological processes, including improving the texture of fermented foods. They can have health benefits, such as immunomodulation and cholesterol-lowering processes [11]. EPSs can play a role in stimulating the immune system, enhancing its response to pathogens. It has been suggested that some specific EPSs can stimulate the production of pro-inflammatory cytokines such as IL-12 and TNF-α, suggesting in vitro immune modulation [19]. It was proposed that EPSs possess antioxidant properties, involve the scavenging of free radicals, and reduce oxidative stress, including superoxide and hydroxyl scavenging [19]. It was suggested that EPSs can bind to cholesterol and, due to this, can improve its removal from the body. Via the mentioned mechanisms, EPSs can contribute to the maintenance of healthy cholesterol levels [19]. Antidiabetic potentials were suggested for some EPSs, associated with their α-glucosidase inhibition, which can assist in managing blood sugar levels, making them potentially beneficial for individuals with diabetes [19]. Antitumor properties are indicated for EPSs produced by LABs, which are still unexplored and deserve additional research attention since they open doors for pharmaceutical applications [20]. As part of controlling spoilage and pathogenic microorganisms, especially in the food industry, the anti-biofilm activity of EPSs is of particular interest. EPSs can interfere with the formation of biofilms by pathogenic bacteria, which is important for preventing infections [21]. EPSs can be considered excellent prebiotics and can be actively involved in promoting the growth of probiotic strains in the gut and directly and indirectly contributing to maintaining healthy microbiota [19]. All mentioned health benefits point to EPSs as promising metabolites, including being a component of functional foods and nutraceuticals [22].

The production of polysaccharides by LABs can play an essential technological role in preparing meat products. Polysaccharides are crucial for improving fermented meat products’ quality, stability, and nutritional value. A key role is related to the specificity of the texture, where polysaccharides act as thickeners and gelling agents, enhancing the texture and mouthfeel of fermented meats. Moreover, polysaccharides are involved in binding water and fat, improving the product’s overall consistency and juiciness. Moreover, polysaccharides may serve as stabilizers, preventing the separation of ingredients and ensuring a uniform appearance of the final product. This is particularly important in maintaining the quality and appearance of fermented meats over time [23]. The role of polysaccharides as prebiotics needs to be acknowledged since they can be considered a source of energy for beneficial bacteria involved in the fermentation process. This can enhance the growth and activity of these bacteria, leading to a more efficient and controlled fermentation [24].

Due to microorganisms’ metabolic properties, polysaccharides can be incorporated into fermented food products to improve the nutritional profile of fermented meats. Polysaccharides can add dietary fiber and other bioactive compounds that contribute to the health benefits of the final product [25]. Moreover, polysaccharides can influence the flavor and sensory properties of fermented meats. They can help develop desirable flavors and aromas, making the product more appealing to consumers [26].

## 4. Bacteriocins

**Figure 3:**
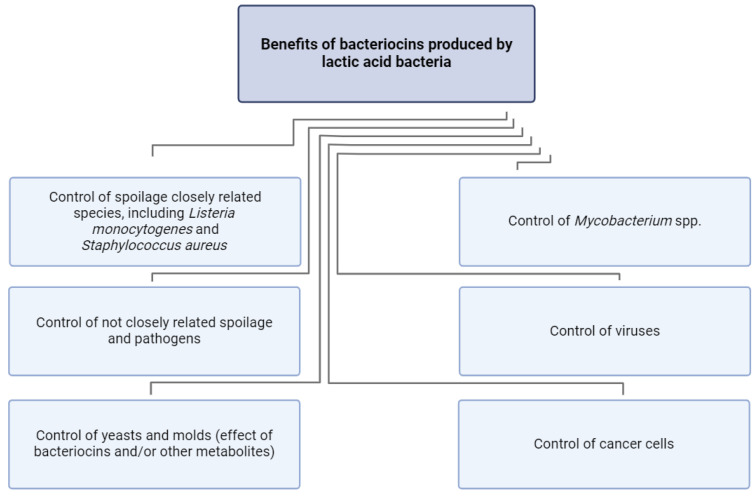
Some of the areas of application of bacteriocins produced by lactic acid bacteria. From simple killing metabolites closely related to the producer’s spoilage and pathogens to potential sophisticated pharmaceuticals with application in the control of viruses, *Mycobacterium* spp., and cancer cells.

Bacteriocins are antimicrobial peptides produced by the ribosomal machinery of LABs (and other types of microorganisms). They are generally described as having inhibitory activities versus other closely related bacterial species [27]. However, it was suggested that some of the bacteriocins produced by LABs could show inhibitory activity against distant microbial species, including Gram-negative bacteria, yeasts, fungus, *Mycobacterium* spp., and viruses [28]. Moreover, it was suggested that some bacteriocins could be involved in quorum-sensing bacterial interactions [29] or even inhibit some cancer cells [30]. Bacteriocins were extensively explored regarding their applications as natural preservatives in food to extend shelf life and enhance the safety of food products, including fermented meat products [7,31]. In the last decades, they have been suggested as potential additional pharmaceutical agents versus some infectious diseases or synergistic co-factors for improving antibiotic efficacy [32] (Figure 3).

Bacteriocin can be considered as an alternative to chemical preservatives in foods, reducing the addition of chemical preservatives and the intensity of heat treatments, resulting in food products that are more naturally preserved and richer in organoleptic and nutritional properties [33]. Some authors have suggested that bacteriocins can be incorporated into packaging material [34]. Consequently, bacteriocins can provide an additional layer of protection against microbial contamination. This can help extend the shelf life of packaged foods [35]. Silva et al. [36] evaluated the antibacterial activity against *Listeria monocytogenes* of packaging containing enterocin. The results were positive, as it was possible to reduce contamination and prevent the migration of the pathogen to the product.

Some bacteriocins have health benefits beyond food preservation, such as boosting the immune system and possessing anticancer properties [35]. Kaur and Kaur [30] suggested that specific interactions between bacteriocins and cancer cells can be further investigated as a perspective for developing new approaches for controlling some specific types of cancer. From this point of view, the role of food products, including fermented meat products, can be regarded as vectors for delivery to the consumers of probiotics (as life organisms), postbiotics (killed probiotics), and beneficial metabolites produced by LABs, including bacteriocins. Moreover, bacteriocins can control antimicrobial resistance in specific food-borne pathogens, making them a valuable tool for food safety [35]. Bacteriocins are versatile compounds (multitasking metabolites) that not only preserve food by inhibiting spoilage and pathogenic microorganisms but also contribute to the health benefits of the food products.

The safety of using bacteriocins in food products is well recognized, and they are considered a safe alternative to traditional chemical preservatives [37,38]. Nisin is classified as GRAS by food safety authorities due to its natural origin and history of safe use in foods [30]. Bacteriocins typically have a very specific mode of action against target microorganisms, defined by their narrow spectrum of activity, which reduces the risk of affecting beneficial bacteria in food products [27,28]. Unlike antibiotics, the use of bacteriocins in food preservation has still not been associated with the development of resistance among pathogenic and food spoilage bacteria [39]. Studies have shown that bacteriocins do not exhibit toxicity toward human cells, making them a safe preservative option [40]. All these mentioned factors endorse them as suitable choices for natural food preservation [35,39,40].

LABs ferment food products are typically naturally enriched by bacteriocins during the food preparation process. In addition to dairy products, fish and seafood, fruits and vegetables, meat products are some of the well-studied fermented food products where bacteriocins have been investigated as contributors to food safety and control of spoilage and food-borne pathogens [40,41].

LABs and their bacteriocins have gained considerable scientific attention for their ability to preserve meat products while preventing spoilage. These compounds can be applied as natural antimicrobial agents rather than chemical preservatives and heat treatment, which can harm meat’s nutritional and sensory qualities. This perspective can be further explored since current trends in society, industry, and academia seek to replace traditional preservation methods that employ common chemical agents. LABs are the principal source of these antimicrobial peptides, and many studies have already documented their promising and convincing features [42,43,44]. The narrow spectrum of activity from these extracellularly released molecules is an interesting element since its inhibition only affects specific microorganisms as pathogens, allowing bacteria related to the fermentation process to work [38,45,46]. Although some of them, such as nisin (the most popular class I bacteriocin), are GRAS by the U.S. Food and Drug Administration (FDA) it is important to highlight that they can also be stable in a variety of conditions such as pH, temperature, presence of lipids, proteins, and other chemical substances. These physical/chemical characteristics can allow them to be industrially applicable. They can be compared with antibiotics, but bacteriocins are more tolerant to higher thermal levels and more active at a wider pH range than traditional antibiotics. Moreover, their primary metabolite nature (ribosomal synthesized) makes them easily suitable for bioengineering, modulating their bioactivity [47], although their proteinaceous nature makes them easily degraded by proteases [48].

Additional research activities are still necessary because only a few bacteriocin preparations, mainly nisin, have reached the market, principally in the dairy industry [49]. Furthermore, there is a lack of evidence regarding their utilization in meat preservation [50]. Another research even showed nisin with limited applicability in meat matrices compared with dairy products [51] and the principal hypothesis that is, in the same way nisin strongly interacts to lipid II, the presence of other kinds of lipids in these meat matrices form complexes decreasing its effect.

However, using other bacteriocins in meat-fermented products can be promising, as shown by Ananou et al. [52]. In this case, it was possible to prove that the bacteriostatic effect of these peptides can be even better when compared with in vitro results. Combined with some intrinsic conditions naturally present in the fermented meat, such as high hydrostatic pressure, the presence of other chemical preservatives and low pH, in addition to processing conditions such as heat treatment, seems to be the way to improve bacteriocins’ effects. Ananou et al. [52] concluded that bacteriocin applied to fermented sausages had a very effective inhibition against *L. monocytogenes* and a slight effect on *Salmonella* at the end of the ripening process—a better result when compared with the in vitro for both bacteria. A very similar result was also reported by Chakchouk-Mtibaa et al. [53] and Darbandi et al. [54].

Considering the mechanism of action of bacteriocins that depends on their capacity to go through the outer membrane (cell wall) and to interact with the plasmatic membrane, numerous research has evaluated the synergistic effect on some compounds such as essential oils [55], plant extracts [56], modified atmosphere packing [57], organic acids [58], and chelators like nitrates, citrates, and EDTA [59]. Each of these interactions showed, in their respective studies, an improvement in the inhibition effects against pathogens. All the mechanisms have not yet been understood. However, in some cases, it is clear that a low pH improves the solubility of bacteriocins, and their combinations with other compounds purely facilitate their interaction at its activation site. Thus, hurdle technologies and the fermented meat environment seem to be the perfect combination for bacteriocins to do their work since this ambiance naturally can contain all these cited elements. So, we must think, is it not worth reconsidering the use of nisin in synergy with other elements? Perhaps more work in this field would be necessary.

In the last two decades, many efforts have been made to prove the effectiveness of the direct application of live cultures of LABs, or purified or semi-purified bacteriocins in meat or meat products. The strategies can vary between direct inoculation during manufacturing or the progressive release of antimicrobial components in primary packaging such as coatings or films [60]. Casquete et al. [42] worked with a treatment of *Latilactobacillus sakei* ST153 culture in combination with the modified atmosphere in cured smoked pork loin stored at 5 °C for 124 days, and results showed a 5 log reduction of *Listeria* spp. for 120 days. De Azevedo et al. [43] tested bacteriocin-like inhibitory substances (BLISs) from *Pediococcus pentosaceus* ATCC 43200 in artificially contaminated RTE pork ham and showed an inhibition of *Listeria seeligeri* NCTC11289 for 6 days. Casaburi et al. [61] assayed sakacins X, T, and P from *Latilactobacillus curvatus* 54M16 in fermented sausage, reducing staphylococci and *Enterobacteriaceae* microbial counts when compared with the control. De Castilho et al. [62] evaluated *Ltb. curvatus* UFV-NPAC1 and its partially purified bacteriocins in fresh pork sausage stored at 7 °C for 10 days, confirming effectiveness on the control (*L. monocytogenes*)’s growth. Barbosa et al. [63] incorporated MBSa2 (*Ltb. curvatus*) bacteriocin as the ingredient in Italian type salami, reaching a 2 and 1.5 log CFU/g reduction on *Listeria* counts after 10 and 20 days, respectively. Castro et al. [64] inoculated Pediocin bacHA-6111-2 (*Pediococcus acidilactici* HA6111-2) as the ingredient in combination with HHP (300 MPa) in Portuguese semidry fermented sausage (alheira), observing a synergistic effect on *Listeria innocua*. Giello et al. [65] evaluated the antilisterial (versus *L. innocua*), antimicrobial, and antioxidant effects of pediocin in refrigerated raw goat meat emulsion, finding that the activity of pediocin can be comparable to the nitrite in raw goat meat emulsion.

Finally, we must point out that bacteriocinogenic LAB live cultures have also been naturally isolated from meat and fermented meat products. These cultures have biopreservative properties and positively affect organoleptic properties [66,67,68,69]. So, we must consider that the isolation of LAB in these products, followed by their safety and bacteriocinogenic characterization, is also a solid strategy for further study and adoption in the coming years.

There is no doubt that bacteriocins are effective against the *Listeria* genus, and all the research listed above confirms this hypothesis. Now we must question, is it sufficient to see this natural preservative in the markets being applied to meat and meat products? Is more evidence needed? Is the narrow spectrum the problem, putting bacteriocins aside? We do not yet know, but we can conclude that the bacteriostatic/bacteriocidal activity of bacteriocins depends on, among other things, other substances that can be present in fermented meat products and, in a synergistic way, improve the bacteriocin’s effects through cell wall and plasmatic membrane interactions. The results showed that class II bacteriocins, like pediocins, have the best potential considering their mechanism of action compared with nisin.

However, while using bacteriocins in meat products is beneficial for food preservation, it has certain limitations. One of the principal problems with industrial applications is the relatively high cost of isolation and purification, which may limit their widespread application in the meat industry [70].

Most bacteriocins have a very specific spectrum of activity, and this narrow inhibitory spectrum can be regarded as beneficial since it does not affect the starter cultures applied. However, some bacteriocins have a limited range of activity, targeting only specific bacteria, which can be considered a limitation from an industrial point of view. This means they may not be effective against all food spoilage or pathogenic microorganisms in meat products [71].

Due to being polypeptides, bacteriocins can be degraded by naturally presented proteolytic enzymes present in meat, reducing their effectiveness as preservatives [70]. However, modification of the N terminal of the bacteriocins by some carbohydrates can improve bacteriocin stability to the same effect as proteases and their stability [72]. Nisin and several other bacteriocins show a high affinity to the lipid II receptor as part of their bactericidal mode of action [27]. This affinity to lipids can be a factor that may reduce bacteriocin activity, since they may interact with lipids from the meat products [73]. Moreover, the efficacy of bacteriocins can be affected by the complex environment of meat products, including factors like pH, fat content, and other compounds [70]. Even being described as particular killing metabolites, bacteriocins, in some cases, may also inhibit the beneficial starter cultures used in the fermentation of meat products, which could negatively impact the desired fermentation process [74]. The hydrophobic nature of some bacteriocins may cause them to partition in the fat phase of meat, which could limit their antimicrobial activity in the aqueous phase where it is needed [71]. These limitations highlight the need for careful consideration and optimization of bacteriocin use in meat products to ensure their effective and economical application as food preservatives.

Research projects have tried to overcome the limitations of using bacteriocins in meat products, and several strategies have been used to enhance their effectiveness and practicality. Some improvements to the purification methods have been suggested. Advancements in downstream purification schemes can help reduce the costs and increase the yield of bacteriocins, making them more economically viable for meat products [75]. This is why, when optimizing and reducing the cost, some authors suggest that starter cultures applied in the fermentation processes will be positive if they can produce bacteriocins [76]. Another option is if bacteriocins can be applied as semi-purified preparations, where residues of the culturing media will not conflict with the final fermented product composition [77,78].

Genetic engineering and biotechnological methods can modify bacteriocins or combine them with other antimicrobial agents to broaden their inhibitory spectrum [50]. Moreover, formulating bacteriocins with stabilizers or protectants or incorporating them into active films and coatings can protect them from degradation and enhance their stability in the complex meat matrix [78,79]. Investigation of the optimal concentration and application methods can ensure that bacteriocins are used effectively without inhibiting desirable starter cultures [50]. Applying bacteriocins in combination with other preservation methods, such as modified atmosphere packaging or natural extracts with antimicrobial properties, can create a synergistic effect that enhances food safety [78]. It has been suggested that bacteriocins can be combined with specific pHs and other antimicrobials where synergistic interaction can enhance the bacteriocins and other antimicrobial activity versus food-borne and spoilage microorganisms [79]. Bacteriocins can be easily degraded by proteolytic enzymes in the gastrointestinal tract [35] and can also be affected by adverse conditions during the food process. Despite this, the encapsulation of bacteriocins may improve their stability and protect them from adverse conditions. One of the principal advantages of encapsulation is that it protects bacteriocins from unfavorable environmental conditions, including pH changes, heat, and particularly the presence of proteolytic enzymes [80].

Additionally, when encapsulated, bacteriocins can be released for a long period. Encapsulation allows for the controlled release of bacteriocins at the target site, ensuring they are active where and when needed [75,80], particularly useful in complex food matrices like meat products [80]. Increased stability is an essential advantage, since incorporating bacteriocins in caring matrixes directly influences their stability in a positive direction and also extends the shelf life of food products by maintaining their antimicrobial activity over a longer period [80]. Encapsulated bacteriocins can be incorporated into various forms, such as films, coatings, liposomes, nanofibers, and nanoparticles, making them versatile for different food preservation applications. The scientific progress in encapsulation techniques has advanced significantly, and various methods have been developed to encapsulate bacteriocins, each with its advantages for food and pharmaceutical applications [80].

Some examples of incorporating bacteriocins include film coatings incorporated into liposomes, which are small vesicles made from the same material as cell membranes. Liposomes are increasingly being used in food preservation due to their unique properties, which enhance the stability and delivery of bioactive compounds [80]. Some of the advantages of the use of liposomes as delivery vectors for bacteriocins include their amphiphilic nature. Liposomes are spherical bilayer structures that can encapsulate hydrophilic and hydrophobic substances, making them suitable for a wide range of food ingredients, including antioxidants and antimicrobials [81]. Liposomes can actively protect encapsulated compounds, protecting bacteriocins from degradation due to environmental factors such as heat, light, and oxygen [81].

Moreover, they can contribute to the bacteriocin’s controlled release; therefore, liposomes can be beneficial for maintaining the desired level of bacteriocins over an extended period [81]. Enhanced bioavailability must be acknowledged as another benefit regarding the use of liposomes, since the encapsulation can improve the bioavailability of certain compounds, making them more effective at lower concentrations [82]. Considering all the benefits mentioned, it is clear why liposomes have been applied in meat preservation to improve antioxidant and antibacterial effects to help extend the shelf life of meat products. In addition, liposomes are considered safe for use in food products as they are made from substances that are GRAS and biodegradable [81]. The use of liposomes in food preservation is a promising area of research and development, offering a natural and effective way to extend the shelf life of food products while maintaining their quality and safety.

Similar advantages have been suggested for the incorporation of bacteriocins into nanofibers. Nanofibers are fine fibers that can encapsulate bacteriocins and release them slowly, providing sustained antimicrobial effects and benefits when applied in biopreservation processes and even as pharmaceutical preparations [80]. Nanofibers are an innovative technology in food preservation, offering new ways to enhance food products’ shelf life and safety. Nanofibers can be used to create edible coatings for perishable fruits and vegetables. In addition to carrying the incorporated bacteriocins, these coatings can slow down package respiration rates, reduce water loss, and decrease oxidation processes, thereby extending shelf life. In addition, nanofibers can improve the barrier properties of packaging materials, protecting food from oxygen, moisture, and microbial contamination [83]. When bacteriomes are incorporated into the nanofibers, this can be associated with slow release, limiting or delaying microbial growth on the food surface, reducing spoilage, and extending the product’s shelf life [29,84]. The existing technology permits the incorporation of various additives into nanofibers, such as antioxidants and flavoring compounds, enhancing their functionalities for food preservation [85], where nanofibers can be regarded as an option for the broader application of nanotechnology in food packaging, which includes the development of active packaging that combines barrier characteristics with antimicrobial agents [84]. Nanofibers in food preservation are a part of the innovative nanotechnology in food packaging, providing a sustainable and practical approach to maintaining food quality and safety.

Moreover, bacteriocins can be incorporated into different nanoparticles, which can protect bioactive components from degradation and allow for targeted delivery to specific sites within the food matrix [80,86]. Nanoparticles have emerged as a promising technology in food preservation, offering innovative ways to enhance food safety and extend shelf life. Nanoparticles can be engineered to have antimicrobial properties, which are effective against a wide range of food-borne pathogens. Metal oxide nanoparticles, such as zinc oxide and titanium dioxide, are commonly used for their antimicrobial activity in food materials [82,87]. They can be designed as controlled release systems, where nanoparticles can act as carriers for enzymes, bacteriocins, antioxidants, anti-browning agents, and other bioactive materials, improving the shelf life of food even after the package is opened [88]. The encapsulation of bioactive components into nanoparticles can improve and extend their efficiency in food preservation. This allows for using lower concentrations of preservatives while maintaining their effectiveness [86,87]. Nanoparticles can be applied directly or incorporated into packaging materials to increase durability, monitor bacterial growth conditions, and improve food’s taste and nutritional absorption [89,90]. However, when using nanoparticles in food preservation, it is essential to ensure they are biocompatible and non-toxic for humans. When some metals are applied, their toxicity and potential side effects must be considered [86,87]. These encapsulation methods are frequently used in food protection, with a significant portion of research also conducted on their potential in the food industry and pharmaceutical applications, particularly for multidrug-resistant therapy [80]. These strategies can help address the challenges of using bacteriocins in meat products, leading to more effective and sustainable food preservation methods.

## 5. Antioxidant Properties

LABs can be effective contributors in the reduction of the negative effects of free radicals associated with their antioxidant properties and reduce the oxidative stress in the body by neutralizing harmful reactive oxygen species (ROS) and reactive nitrogen species (RNS) [91,92]. LABs possess enzymatic machinery that contributes to their antioxidant properties, including superoxide dismutase and NADH peroxidase. These enzymes can be involved in the breaking down of ROS and protecting cells from oxidative damage [92]. Moreover, when consumed as probiotics, LABs can enhance the body’s antioxidant defenses and improve the reduction of inflammation and prevent oxidative stress-related diseases like cardiovascular diseases and certain types of cancers [92,93]. Associated with conducting fermentation processes and being involved in the preparation of fermented food products, LABs can contribute to the increase in the antioxidant capacity of foods. This is due to the production of bioactive compounds that have antioxidant properties, such as specific organic acids, peptides, and exopolysaccharides [91]. Moreover, LABs with strong antioxidant properties are being explored for their potential in preventing and managing oxidative stress-related conditions. They are also being studied for their role in promoting gut health and overall well-being [93].

Enzymes involved in the antioxidant properties of LABs are crucial for protecting the bacteria and the host organism from the damaging effects of ROS. NADH oxidase is the enzyme associated with reducing oxygen to water, thereby minimizing the formation of superoxide radicals [92]. However, NADH peroxidase is related to reducing hydrogen peroxide in water, which is important for protecting the cell from oxidative damage caused by hydrogen peroxide. Moreover, superoxide dismutase (SOD) is the enzyme that catalyzes the dismutation of superoxide radicals into oxygen and hydrogen peroxide, which is then further reduced by catalase/pseudocatalase or peroxidase [92]. Regarding thioredoxin reductase, the process is associated with the regeneration of thioredoxin. This protein acts as an antioxidant by facilitating the reduction of other proteins by cysteine thiol–disulfide exchange. The mentioned enzymes are only a few examples related to enhancing the antioxidant capacity of LABs, enabling them to survive in oxidative environments and potentially confer health benefits when used as probiotics. Moreover, they can protect the host from the toxic effects of ROS and contribute to the prevention of various diseases related to oxidative stress [92].

Bryukhanov et al. [92] reported different LABs regarding their antioxidant properties with the objective of their application in fermentation processes for preparing various food products, including meat. Since LABs do not possess a complete electron transport chain, it was previously believed that they could not exist under oxygenic conditions. However, several LAB strains were observed to possess principal antioxidant defense enzymes with high specific activities. More specifically, it was reported that there can be two types of catalases and NADH peroxidases. Heme-containing catalase is synthesized in many species of lactobacilli when heme or hematin is present in the culture medium (often reposted as pseudo-catalase activity). Catalases of the second type (Mn-containing) are also present in the cells of some lactobacilli species. From a technological point of view (starter and adjunct cultures or probiotics), LAB cultures with pronounced antioxidant activity are of high scientific and commercial interest since they are potentially able to protect the host organism from the toxic effects of ROS and contribute to the prevention of cardiovascular, inflammatory, and oncologic diseases [92].

Kaveh et al. [94] reviewed biopreservation, which implies the application of microorganisms or their metabolites to extend the shelf life of food products. Focus was given to LABs regarding fermentation processes and their application in the biopreservation of meat and meat products. The outcome pointed out by the authors was the high potential of various LAB strains and their metabolites, not only bacteriocins as biopreservatives, but antioxidant properties of beneficial strains in meat and meat products for extending their shelf life. In this regard, their combined use with other novel technologies or natural antibacterial compounds as a hurdle technology is a more effective method to compete with synthetic preservatives [94].

It has been suggested that pediococci and lactobacilli could actively contribute via their antioxidant properties to the quality of fermented meat products. For example, it can be pointed out that strains of *P. pentosaceus* can significantly reduce the lipid and protein oxidation in sausages, suggesting their use as antioxidant agents in the production of fermented meat products [95]. Moreover, *Lactiplantibacillus plantarum* strains can prevent fat oxidation and myoglobin oxidation in sausages, contributing to better color and reduced nitrite residues [96]. These works demonstrate the significant role of LABs in meat fermentation, not only for product quality and safety but also for their antioxidant properties, which can be beneficial in reducing oxidation in meat products and potentially improving human health.

## 6. Reduction of Chemical Additives

Associated with their metabolic capacity, LABs can be applied as an alternative to some chemical additives in food production, particularly in meat and dairy products. As producers of arsenal of natural preservatives (organic acids, such as lactic acid and acetic acid) during fermentation by LABs applied as starter or adjunct culture, they can effectively improve safety processes and, at the same time, cope with the demand of the consumers for more natural food products free of synthetic chemical additives. These acids lower the pH of the food, creating an environment that inhibits the growth of spoilage organisms and food-borne pathogens [97].

Moreover, LABs can enhance the flavor profile of foods, reducing the need for artificial flavor enhancers due to the production of volatile compounds during fermentation, such as diacetyl, which imparts a buttery flavor to dairy products or can be involved in the biotransformation of some phenolic compounds and, as a result, actively contributes to the formation of specific organoleptic characteristics for meat products [98]. In biopreservation processes, an active role is associated with producing bacteriocins, a proteic compound produced by different LABs, which can target specific pathogens. Moreover, bacteriocins are effective in small quantities and can replace chemical preservatives to ensure food safety [99]. By competitive exclusion, bacteriocins can contribute to the reduced need for chemical additives typically used to control microbial growth [98]. The presence of LABs can positively contribute to increasing the nutritional value of food by producing vitamins and bioactive peptides, reducing the need for chemical additives, and producing more natural and clean-label products [97,98,99,100].

With the advancement of scientific research, it has been discovered that not only live probiotics cells can generate health benefits but also the metabolites generated by them, or even death cells, called postbiotics [101,102]. One of the main advantages, for example, is the lack of need for storage and transportation in a refrigerated environment, which reduces costs for the industry, in addition to not interacting with the food matrix. Regarding this concept, killed microbial preparation has been suggested as beneficial, and appropriate scientific arguments have been provided [102].

Regarding the nomenclature, it is important to mention that “fermented”, “contains live cultures”, or specific names of LAB strains have to be present on the package and present some differences. Fermented food products can contain live bacterial cultures, but their numbers can be lower to be considered as a probiotic product (more than 6 log CFU/g). Moreover, not all live bacterial cultures are considered probiotics. Even if it is beneficial for the fermentation process, to be considered a probiotic, the bacterial culture needs to be proven to be safe, free of virulence factors and antibiotic resistance genes, and provide beneficial properties for the host, confirmed by appropriate animal and human trials studies [101].

Several meat products are naturally fermented, and LABs involved in the fermentation processes can be presented in final products. Some of them may even have beneficial effects for the consumers. This is the background to the development of fermented meat products with probiotics properties, where meat products can be used as a vector for the delivery of probiotic strains [103].

## 7. Biotechnological and Technological Properties of LABs in Fermented Meat Products

The indigenous microbiota of meat products is diverse and comprises bacteria, filamentous fungi, and yeasts, with LABs being the most commonly found among the bacteria, with their initial indigenous population varying between 3 and 5 logs CFU/g [104,105]. During the ripening process, the LAB population becomes dominant in fermented meat, reaching values exceeding 6 to 8 logs CFU/g due to their optimal adaptation to the ripening environment of these meat products, which actively contributes to the formation of the flavor, test, structure, and biotransformation of proteins, lipids, and carbohydrates [105]. The complex role of LABs in the fermentation processes is related to technological, biopreservative, and organoleptic benefits [44]. LABs are crucial in this type of product as they promote important and desirable characteristics, since they are not only capable of converting sugars into organic acids but also possess a range of enzymes capable of breaking down proteins, fats, and carbohydrates into smaller compounds responsible for producing desirable flavors, aromas, and textures [96]. However, excess production of acids (including lactic acid), biotransformation of proteins, or production of biogenic amines (decarboxylation of some amino-acids) can be processes associated with the metabolic specificity of some LABs. On such occasions, they can be considered as spoilage. Thus, the appropriate evaluation for any new bacterial culture with an intense application as a starter (or adjunct) culture in food fermentation processes must be detailed and evaluated for potential negative effects [12,67]. Several examples regarding the production of antimicrobial peptides and other antimicrobials have already been mentioned and discussed in the previous chapters of the current work, emphasizing the role of LABs in the safety of fermented meat products.

The most commonly recorded indigenous LAB microbiota species are *Ltb. sakei*, *Ltb. curvatus*, and *Lpb. plantarum*. The most common genera in fermented sausages are *Lactobacillus* (in the context of the meaning before changes in taxonomy in 2020 [1]), *Pediococcus*, *Leuconostoc*, *Weisella*, and *Enterococcus*. During ripening, these microorganisms play an important role as they produce lactic acid, promoting acidification of the environment, and bacteriocins, making the environment unsuitable for spoilage bacteria, such as *Pseudomonas* spp. and enterobacteria [104].

Starter cultures composed of LABs are responsible for improving the product’s flavor, color, appearance, texture, and juiciness, along with promoting its safety until the end of its shelf life. Characteristics such as accelerating the fermentation process, producing flavor, texture, reducing nitrate to nitrite, good growth at different temperatures, homofermentative metabolism, no formation of peroxides, survival ability during ripening, antagonism against pathogens and spoilage microorganisms, and having a good cost/benefit ratio are important and desirable for LABs used in the fermentation processes of meat products [104,106,107]. According to the European Food Safety Authority [108], safety factors such as antibiotic resistance profile, bacteriocin production, and absence of amino-acid decarboxylation activity should be considered when selecting cultures with biotechnological potential for application in fermented products.

LABs, commonly used as starter cultures in meat products, are mostly homofermentative, resulting in the exclusive production of lactic acid as a fermentation by-product. Acidification directly impacts the sensory quality of the product by imparting a “tangy” flavor, and the pH drop helps in the coagulation of meat proteins and in promoting coloring reactions, for example [105]. Flavor formation is primarily related to lactic acid production. However, other products from heterofermentation may also be related to these product characteristics, such as acetic acid, ethanol, CO_2_, and pyruvic acid [104], meaning LABs are responsible for carbohydrate metabolism, proteolysis of amino acids, and lipolysis of free fatty acids [105].

LABs produce various organic acids, such as lactic and acetic, during fermentation through glycolysis. The formation of other compounds depends on various factors, including the type of bacteria present, temperature, and the composition and quantity of available carbohydrates. There are two main pathways of lactic fermentation: homofermentative LABs, which follow the glycolysis pathway, known as the Embden–Meyerhof–Parnas’s pathway, and heterofermentative LABs, which follow the 6-phosphogluconate/phospho-ketolase pathway. Sucrose is rapidly hydrolyzed and fully utilized during fermentation, resulting in a less pronounced acidic flavor than dextrose despite reaching equivalent final pH values. In the fermentative process, lactic acid production by LABs reduces pH, which is crucial for product quality. The pH can decrease in dry sausages from 5.8 to 5.4, while in semi-dry sausages, it can vary from 4.8 to 4.6 [96]. It is important to note that the acidification process in meat products should occur gradually, as a rapid pH decrease leads to extensive protein denaturation, rendering the product unacceptable [109].

During ripening, aromatic compounds are formed by microorganisms due to the catalysis of amino acids and small peptides [106]. This process is known as proteolysis, where the hydrolysis of myofibrillar and sarcoplasmic proteins begins by the action of endogenous enzymes and, subsequently, bacterial proteases and peptidases during ripening, and this degradation influences the consistency of the product as small peptides and amino acids are formed [105,110]. It is important to note that the proteolysis process is influenced by temperature, pH, product formulation, processing conditions, and type of microbial culture [104].

Lactobacilli are the most reported LABs in meat products. Other than lactic acid, they can produce ethanol, CO_2_, ketones, aldehydes, furans, and bacteriocinogenic compounds. *Ltb. sakei*, *Ltb. curvatus*, and *Lpb. plantarum* are the most technologically relevant and widely used as starters in this type of product [108]. According to Kröckel [106], *Ltb. sakei* and *Ltb. curvatus* are the most predominant LABs in dry-fermented sausages. Species such as *Companilactobacillus versmoldensis*, *Lpb. plantarum*, *Levilactobacillus brevis*, *Companilactobacillus farciminis*, *Companilactobacillus alimentarius*, *Weissella*, pediococci, and leuconostoc are found in these products but in less numbers [111,112,113,114,115].

Pediococci are also commonly used LABs in the fermentation of meat products because they contribute to the acidification process and to the production of other compounds such as acetic acid, acetoin, CO_2_, and ethanol, along with initiating the production of aromatic compounds from protein precursors [104,106]. The species most used as starters are *P. acidilactici* and *P. pentosaceus* [104,106].

Therefore, LABs in fermented products are essential to improve and achieve the desired sensory characteristics. In addition to contributing to aroma, flavor, and texture, these bacteria also play a crucial role in product standardization and microbiological safety, while providing nutritional characteristics.

## 8. Enrichment with GABA

GABA (gamma-aminobutyric acid) is considered the brain’s primary inhibitory neurotransmitter, which means it helps to reduce neuronal excitability throughout the nervous system. Essentially, GABA acts like a brake to slow down brain activity, producing a calming effect. Fermented foods are considered as one of the natural sources for GABA, related to the fact that certain bacteria involved in the fermentation process can actively produce GABA [116,117]. Several studies have confirmed that LABs are widely studied and known due to their application in numerous food products. LABs possess the ability to produce different metabolites such as short-chain fatty acids, lactic acid, bacteriocins, γ-aminobutyric acid, and others. Beyond their importance in the food industry, researchers have extensively explored LABs for their diverse applications [116].

The enzyme glutamate decarboxylase (GAD), present in microorganisms, animals, and plants, promotes the synthesis of GABA by decarboxylating L-glutamate [117]. According to different studies, GABA contributes to various health benefits. This neurotransmitter plays a role in anti-hypertensive processes [118], antidepressant effects [119], prevention of neurological diseases [120], antidiabetic effects [121], anticancer properties [122], and other pathological processes.

In a study conducted by Kuda et al. [123], they discovered the presence of GABA in “aji-no-susu”, a typical fermented product from Japan made of fish and cooked rice widely known as narezushi, after LABs fermentation. The study utilized 12 different types of “aji-no-susu” collected from 7 distinct retailers and 5 producer families for their own consumption. They isolated 336 strains and selected 4 LAB strains based on their halo-tolerant capacity. Two strains with the highest halo-tolerant capacity were identified as *Loigolactobacillus rennini* (99.2%), and two strains with the highest halo-intolerant capacity were identified as *Lpb. plantarum* (99.9%). It was possible to verify the capacity for GABA synthesis independent of their halo-tolerance.

Kuda et al. [123] found a higher presence of GABA in medium and long-term products (4 and 12 months) after analyzing the content of free amino acids and related compounds. They observed 1.46–1.37 mg g^−1^ in the rice portion and 1.30–1.24 mg g^−1^ in the fish portion, respectively.

Ratanaburee et al. [124] analyzed “nham”, a fermented Thai sausage made from meat (commonly pork-based), which showed beneficial properties in the fermentation process. They selected four strains of LAB highly productive in GABA (>8000 mg L^−1^), classifying three as *P. pentosaceus* (HN8, NH102, and NH116) and one as *Levilactobacillus namurensis* (NH2).

According to the same study, researchers identified GABA-producing LABs in different meats. They selected 14 strains that showed evident activity in GABA production, with 4 strains notable for their high production capacity (>8000 mg L^−1^). Additionally, seven other strains produced levels above (>6000 mg L^−1^), while only one strain had values below <6000 mg L^−1^ (NH84 3506 mg L^−1^) [120]. The authors [124] observed higher GABA production indices in fermented products based on ground pork, followed by beef and fish. Among the 14 isolated strains, 50% originated from ground pork-based products, and of these, 85% exhibited strains with GABA production exceeding 6000 mg L^−1^. They identified the three strains with the highest GABA index as *P. pentosaceus* (HN8, NH102, and NH116), all having a pH similarity of 3.8. They produced 9060, 8411, and 8386 mg L^−1^ of GABA, respectively. The authors also isolated these strains from two types of animal protein, HN8 originating from beef and NH102 and NH116 from ground pork. These findings demonstrate that *P. pentosaceus* is a significant producer of GABA beyond its presence in the food matrix [120]. The analysis conducted by Ratanaburee et al. [124] identified *Lvb. namurensis* (NH2) as a major producer of GABA (7339 mg L^−1^) in a pH of 4.2 in one of nham products made from ground pork. This demonstrates that other strains are also strong candidates for GABA production.

Thuy et al. [125] conducted a study using “ruoc”, a fermented salty shrimp paste. They discovered the presence of strains of lactic acid bacteria responsible for fermenting the food and capable of producing GABA. They isolated four strains that produce GABA (R1, R3, R12, and R13), with strain R13, identified as *Lactiplantibacillus pentosus*, exhibiting the highest GABA production capacity (14.69 mM ± 0.16). The same author, in another analysis, using “man nem”, a fermented fish sauce, isolated six strains of highly GABA-producing lactic acid bacteria named MN2, MN3, MN4, MN5, MN9, and MN12, with production ranging between 8 and 16 mM of GABA among them. Among the isolates, strain MN12, identified as *P. pentosaceus*, was the highest producer of GABA under its optimal conditions, with a quantity of 27.9 ± 0.42 mM, showing that *P. pentosaceus* is a strong GABA-producing LAB [125].

## 9. Other Beneficial Metabolites

LABs can produce various enzymes, including amylase, protease, and lipase, which play a role in the breakdown of starch, proteins, and fats, respectively, during fermentation processes [11]. The role of enzymes is essential from the technological point of view [126,127]. However, they may play a role in safety since they can directly and indirectly inhibit spoilage and food-borne pathogens. For example, Aouadhi et al. [128] evaluated the antimicrobial activity of different LAB strains against *Bacillus sporothermodurans* LTIS27, *Escherichia coli* ATCC 8739, *Salmonella typhimurium* NCTC 6017, *Staphylococcus aureus* ATCC 29213, *Pseudomonas aeruginosa* ATCC 27853, *L. monocytogenes* ATCC 7644, *Bacillus cereus* ATCC 1247, *Bacillus coagulans*, *Enterococcus faecalis* ATCC 29212, and *Klebsiella pneumonia* ATCC 15380. In general, all strains tested were able to produce the following enzymes: esterase, esterase lipase, lipase, valine arylamidase, cystine arylamidase, N-acetyl-β-glucosaminidase, ᾳ-mannosidase, ᾳ-galactosidase, and β-glucuronidase, and were effective even against spores, demonstrating the ability to use these strains in food products.

LABs produce organic acids, such as lactic acid, that can act as antimicrobial agents in food products [129]. Lactic acid is the primary metabolite produced by LABs and is responsible for the acidic environment that preserves food and inhibits the growth of food-borne and spoilage organisms [11]. Bartkiene et al. [130] evaluated the antimicrobial effect of LABs on cold smoked pork sausages’ surfaces, in addition to making a relationship with the production of organic acids. The amount of organic acid increased during fermentation, demonstrating high activity against pathogenic and food spoilage bacteria. Furthermore, in a critical review, Coban [131] reported several studies in which specific concentrations of lactic acid can be used as antimicrobial agents in the meat industry, mainly against important pathogens such as *Salmonella* spp. and *L. monocytogenes*. In addition to lactic acid, LABs can produce acetic acid and other organic acids by sugar fermentation that can affect the intracellular pH homeostasis of the pathogen, which will interfere with essential metabolic reactions [132].

Diacetyl is another metabolite commonly produced by LABs, whose main characteristic is to modify the sensory characteristics of foods, normally associated with a buttery flavor [133]. Despite this, the use of diacetyl as an antimicrobial agent also has a long history in the literature; for example, Kang and Fung [134] already reported its bioprotective action against *E. coli* O157: H7 and *S. typhimurium* in meat products. As mentioned, because it is a metabolite of LABs, it is normally safe for consumption and is normally present in food products, being an important compound in terms of sensory properties and also as a bioprotective agent in meat products [135].

LABs are known for their ability to produce various vitamins, especially those from the B group, which are essential for human health, including those associated with fermented meat products. LABs are involved in synthesizing water-soluble vitamins, including folates (vitamin B9), riboflavin (vitamin B2), and cobalamin (vitamin B12) [136]. Some LAB strains have been characterized as caring genes involved in the biosynthesis of riboflavin [137], and their role in improving the vitamin enrichment of fermented foods has been suggested [138]. It has been suggested that using vitamin-producing LABs could be a cost-effective alternative to vitamin fortification programs and could lead to the creation of novel vitamin-enriched meat products [136].

## 10. Essential Steps in the Selection of LABs for Application in Meat Products

For the safe application of LABs (in fact, any microbial culture) into food products, it is compulsory to have an appropriate identification of the strains. This includes a combination of molecular techniques and biochemical and physiological approaches. Moreover, whole genome sequences are required by several national agencies regarding certification for new microbial cultures applied in food processing [139].

In addition to the technological and biopreservation properties [94], adjunct microbial cultures can bring additional beneficial properties, including the production of vitamins. LABs can be associated with producing bioactive properties, such as cholesterol reduction and antimicrobial activity, making them effective as natural preservatives in innovative food preservation technologies [94]. This can be evaluated by employing microbiological assays or chromatography to quantify the vitamins produced by each LAB strain. It has been suggested that various strains of LABs are isolated from fermented meat products for their ability to produce B-group vitamins like riboflavin (vitamin B2), niacin (vitamin B3), and cobalamin (vitamin B12) [136]. Moreover, the production of vitamins needs to be optimized, including confirming that this will be a realistic process in the meat environment. Different growth conditions to maximize vitamin production, such as varying the temperature, pH, or nutrient composition of the growth medium, are only some variables that need to be evaluated [138]. Applying some appropriate animal models and clinical trials need to be considered when evaluating the potential health benefits of consuming these vitamin-enriched meat products. Such studies will contribute to understanding the functional properties of LABs in meat products and their potential as natural biofortification agents to enhance the nutritional value of foods [136]. LeBlanc et al. [136] reported on the current knowledge of vitamin biosynthesis by LABs and showed how the proper selection of starter cultures and probiotic strains could be useful in preventing clinical and subclinical vitamin deficiencies. They also discussed using genetic engineering strategies to increase vitamin production or create novel vitamin-producing strains [136].

## 11. Conclusions and Perspectives

LABs are an extensively studied group of microorganisms known for their technological benefits in meat and meat products and health-promoting effects. They are essential in the fermentation process of meat products that are responsible for lowering the pH, which inhibits the growth of spoilage and pathogenic microorganism via the production of bacteriocins and other antimicrobials, which are natural antagonists to the spoilage and pathogens that contribute to the extension of the shelf life; contribution to the flavor and texture formation and enhancement, where the metabolic activities of LABs contribute to the development of the complexity of the gastronomic desirable characteristics of the final products. Nutritional benefits, associated with production of vitamins and other bioactive compounds, including reducing cholesterol levels to the degradation of nitrites and provide antioxidant activity, which improves the safety of meat products by reducing harmful compounds and extending shelf life. Overall, LABs play a crucial role in improving the quality, safety, and nutritional value of meat products. The bioactive compounds produced by LABs during their metabolisms exhibit many beneficial properties, including antimicrobial, antioxidant, antihypertensive, immunomodulatory, anti-inflammatory, and potential anticancer effects. However, the virulence potential presented by certain strains of LABs is an essential factor to be considered before application in the food process. Alternatively, even though most LABs have been considered safe based on their long history of applications in fermented food products, strain-based safety assessments must be considered an essential step in implementing new strains in food fermentation processes. Furthermore, it is important to consider all parameters that can harm cell viability during storage. From the studies presented and discussed in this review, it is possible to conclude that LABs have comprehensive functions in meat and meat products and are applied more broadly than the starter culture function. Furthermore, further studies are recommended to validate new formulations and the effects of LABs on quality and safety parameters.

## Data Availability

No new data were created or analyzed in this study. Data sharing is not applicable to this article.
